# Quality of Life and Symptomatology Before and After Nissen Fundoplication

**DOI:** 10.4021/gr216e

**Published:** 2010-07-20

**Authors:** John-Patrick Devine Byars, Kishore Pursnani, Muntzer Mughal

**Affiliations:** a School of Medicine, Manchester University, Oxford Road, Manchester M13 9PT, UK; b Department of General Surgery, Royal Preston Hospital, Sharoe Green Lane, Preston, UK

**Keywords:** Quality of life, Fundoplication, Hiatus hernia, Gastro-esophageal reflux disease (GERD)

## Abstract

**Background:**

Post surgical quality of life (QOL) plays an important role in the decision making process for patients. This study evaluated the subjective opinion of those that underwent Nissen fundoplication to correct their symptoms of hiatus hernia. This study was to evaluate the quality of life and symptomatology before and after in those patients that underwent Nissen fundoplication over an 8-year period.

**Methods:**

A questionnaire that graded the severity of symptoms and quality of life pre- and post-operatively was sent out to those patients that had undergone Nissen fundoplication.

**Results:**

After the operation the symptoms of heartburn, regurgitation, burping and difficulty lying down were markedly decreased (P < 0.0001). There was however an increased incidence of flatulence associated with the procedure (P < 0.0001). Despite this the quality of life was significantly increased in those that underwent Nissen fundoplication (P < 0.0001).

**Conclusions:**

Nissen Fundoplication has a positive impact on quality of life and is effective in reducing symptoms of heartburn, regurgitation, burping and difficulty lying down associated with a hiatus hernia. There is however an increase in the incidence of flatulence associated with the procedure. In spite of this, 94% of patients would recommend the procedure to someone else.

## Introduction

Gastro-esophageal reflux disease (GERD) is the result of spontaneous and involuntary reflux of the contents of the stomach into the oesophagus as a result of an incompetent lower oesophageal sphincter [1]. One of the most common causes of GERD is a hiatus hernia with an incidence in the general population of 5 per 1000 [2]. The history of hiatal hernia surgery dates back to 1919 where Soresi undertook the first elective surgery. Following this operation, surgery witnessed an evolution from the anatomical repair to physiological restoration with Nissen and Belsey developing their famous operations [3].

Laparoscopic Nissen fundoplication (LNF) was first undertaken in 1990 in order to provide short and medium term control of reflux symptoms similar to results seen in open fundoplication [4]. Since then it has gained international praise as being the technique of choice in the surgical correction of GERD associated with hiatus hernia [5].

This study will evaluate the patients’ subjective opinion in quality of life (QOL) changes and symptomatology pre- and post-operatively using a questionnaire.

## Patients and Methods

The patients selected for this study all underwent either laparoscopic Nissen fundoplication (LNF) or open Nissen fundoplication (ONF) in the last 8 years in the Royal Preston Hospital or Chorley and South Ribble Hospital (Lancashire, UK). A questionnaire that assessed the severity of symptoms and quality of life pre- and post-operatively were sent out to all 94 patients. Out of the 55 (58%) that replied, 3 had errors by way of accidently missing a page of questions out and thereby were discounted.

The questionnaire was split into 2 sections, the first referring to the pre-operative symptoms and quality of life and the second section referring to the post-operative symptoms and quality of life. The Visick score was used to assess the degree of heartburn. There were specific questions that evaluated symptoms such as dysphagia, regurgitation, burping, bloatedness, flatulence and difficulty lying down. A question that evaluated the quality of life was also included and lastly a question that assessed as to whether or not the patient would recommend the surgery to someone else. The Visick score was on a scale of 1 to 4 (1 = no heartburn and 4 = severe symptoms requiring medication). The rest of the questions relating to symptomatology were on a scale of 1 to 5 (1 = not having the symptoms at all and 5 = experiencing the symptoms every day). The quality of life question was also on a scale of 1 to 5 (1 = not affected at all and 5 = extremely affected). The last question asked whether or not they would recommend the surgery and was on a yes, no or impartial basis.

## Results

Out of the 52 patients finally included in the final analysis 29 were male and 23 were female. The ages ranged from 27 to 82 with a mean age of 57.

The shortest duration from surgery when the questionnaire was taken was 3 months and the longest was 96 months with a mean of 43 months from surgery.

Forty-nine of the surgical procedures were laparoscopic, 3 were converted to open from laparoscopic and 1 procedure was an open Nissen fundoplication. One procedure had to be surgically corrected at a later date due to post-operative complications.

**Figure 1:**
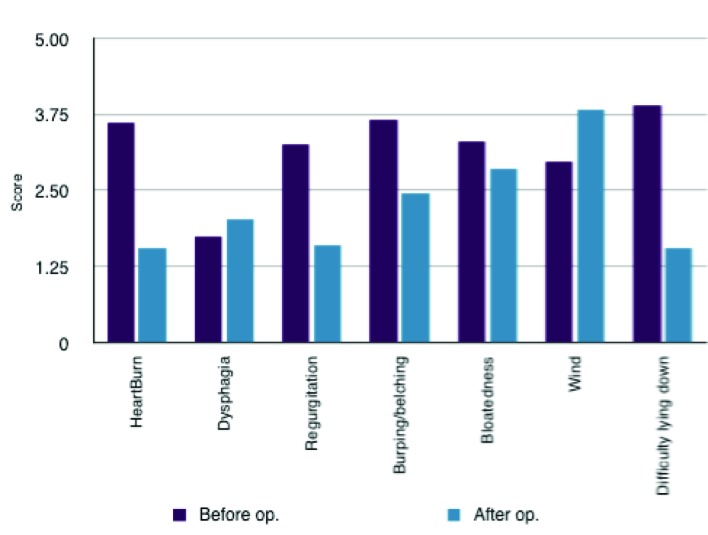
Comparison of symptomatology pre- and post-operatively.

**Figure 2:**
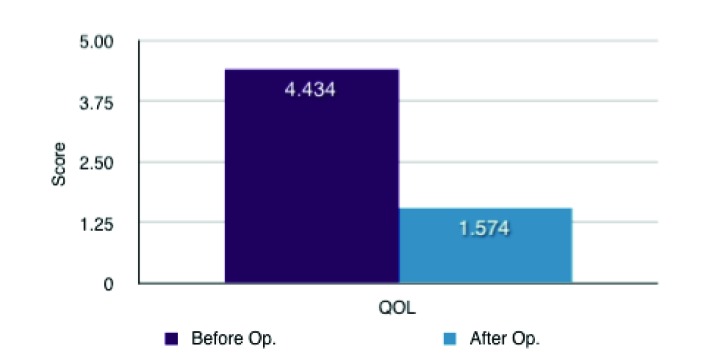
Mean QOL differences (1 = not affected at all and 5 = extremely affected).

The results showed that there was a significant decrease post-operatively in the symptoms of heartburn with a mean difference of 2.057 ± 0.361 (SD) (P < 0.0001). Regurgitation, burping/belching and difficulty lying down were also significantly decreased post-operatively (with mean differences of 1.679 ± 0.515 (SD) (P < 0.0001); 1.208 ± 0.528 (SD) (P < 0.0001); 2.358 ± 0.436 (SD) (P < 0.0001) respectively). There was non-significant differences associated with dysphagia and bloatedness (P = 0.163 and 0.1229 respectively). There was however an increase in flatulence experience by the patients post-surgically with a mean difference of 0.868 ± 0.449 (SD) (P < 0.0001). Nevertheless despite this increase in flatulence there was a substantial subjective improvement in quality of life post-operatively with a mean difference of 2.849 ± 0.399 (SD) (P < 0.0001) (Table 1, Fig. 1 and 2). This increase in quality of life was compounded by 49 (94%) of the patients reporting that they would recommend the surgery to someone else. 2 patients said that they would not recommend the surgery and 1 was impartial.

**Table 1:** Post-operative Changes in Symptoms and Quality of Life for 52 Patients

Symptom	Average score before	Average score after	Mean of difference	C.I. (95%)	P value
Heartburn	3.63	1.566	2.057	(1.696, 2.417)	< 0.0001
Dysphagia	1.759	2.038	0.264	(-0.639, 0.111)	0.163
Regurgitate	3.259	1.623	1.679	(1.164, 2.194)	< 0.0001
Burping/Belching	3.667	2.453	1.208	(0.68, 1.735)	< 0.0001
Bloatedness	3.315	2.868	0.434	(-0.121, 0.989)	0.1229
Flatulence	2.981	3.83	0.868	(0.419, 1.317)	0.0003
Diff. lying down	3.907	1.566	2.358	(1.922, 2.795)	< 0.0001
QOL	4.434	1.574	2.849	(2.450, 3.247)	< 0.0001

## Discussion

Several studies have measured the quality of life following Nissen fundoplication by way of quantifying the success of the procedure [6-8]. Such studies have showed similar significant results with regards to improved QOL. This study has demonstrated that Nissen fundoplication is an effective procedure for the correction of a hiatus hernia. There was substantial reduction in the symptoms of heartburn, regurgitation, burping and difficulty lying down. Symptoms such as dysphagia and bloatedness were not significantly affected and there was an increase in flatulence in patients post-operatively. Despite this there was a significant increase in the quality of life following the procedure.

Retrospective evaluation has its limitations with regards to differing times from the surgery and this could have been improved by a longitudinal study. The study size could also have been increased to include all Nissen fundoplications done within the Trust.

In conclusion, there is a notable decrease in QOL associated with hiatus hernia and as such the increased QOL associated with Nissen fundoplication should play a significant part when informing patients about the procedure.

## References

[1] Chen D, Barber C, McLoughlin P, Thavaneswaran P, Jamieson GG, Maddern GJ (2009). Systematic review of endoscopic treatments for gastro-oesophageal reflux disease. Br J Surg.

[2] Luketich JD, Raja S, Fernando HC, Campbell W, Christie NA, Buenaventura PO, Weigel TL (2000). Laparoscopic repair of giant paraesophageal hernia: 100 consecutive cases. Ann Surg.

[3] Stylopoulos N, Rattner DW (2005). The history of hiatal hernia surgery: from Bowditch to laparoscopy. Ann Surg.

[4] Mahon D, Rhodes M, Decadt B, Hindmarsh A, Lowndes R, Beckingham I, Koo B (2005). Randomized clinical trial of laparoscopic Nissen fundoplication compared with proton-pump inhibitors for treatment of chronic gastro-oesophageal reflux. Br J Surg.

[5] Booth MI, Jones L, Stratford J, Dehn TC (2002). Results of laparoscopic Nissen fundoplication at 2-8 years after surgery. Br J Surg.

[6] Slim K, Bousquet J, Kwiatkowski F, Lescure G, Pezet D, Chipponi J (2000). Quality of life before and after laparoscopic fundoplication. Am J Surg.

[7] Kamolz T, Bammer T, Wykypiel H, Pasiut M, Pointner R (2000). Quality of life and surgical outcome after laparoscopic Nissen and Toupet fundoplication: one-year follow-up. Endoscopy.

[8] Glise H, Hallerback B, Johansson B (1995). Quality-of-life assessments in evaluation of laparoscopic Rosetti fundoplication. Surg Endosc.

